# Knockdown of MCM8 inhibits development and progression of bladder cancer in vitro and in vivo

**DOI:** 10.1186/s12935-021-01948-2

**Published:** 2021-04-30

**Authors:** Wei Zhu, Fei Gao, Hongyi Zhou, Ke Jin, Jianfeng Shao, Zhuoqun Xu

**Affiliations:** 1 Shengli Clinical Medical College of Fujian Medical University, Fuzhou, 350001 China; 2 Department of Urology, Fujian Provincial Hospital, Fuzhou, 350001 China; 3 Wuxi People’s Hospital Affiliated to Nanjing Medical University, 299 Qingyang Rd, Wuxi, 214023 China

**Keywords:** Bladder cancer, MCM8, Cell proliferation, Cell apoptosis, Cell migration

## Abstract

**Background:**

Bladder cancer is a frequently diagnosed urinary system tumor, whose mortality remains rising. Minichromosome maintenance eight homologous recombination repair factor (MCM8), a newly discovered MCM family member, has been shown to be required for DNA replication. Unfortunately, little is known concerning the roles of MCM8 in bladder cancer.

**Methods:**

The present study, we aimed at probing into the impacts and detailed mechanisms of MCM8 in bladder cancer progression. In this study, MCM8 expression level was detected through immunohistochemistry staining (IHC), qRT-PCR and Western blot assay. Silenced MCM8 cell models were constructed by lentivirus transfection. In vitro, the cell proliferation was evaluated by the MTT assay. The wound-healing assay and the transwell assay were utilized to assess the cell migration. Also, the cell apoptosis and the cell cycle were determined by flow cytometry. Moreover, the Human Apoptosis Antibody Array assay was performed to analyze the alterations of apoptosis-related proteins. The in vivo experiments were conducted to verify the effects of MCM8 knockdown on the tumor growth of bladder cancer*.*

**Results:**

The results demonstrated that compared with normal adjacent tissues, MCM8 expression in bladder cancer tissues was strongly up-regulated. The up-regulation of MCM8 expression in bladder cancer may be a valuable independent prognostic indicator. Of note, MCM8 inhibition modulated the malignant phenotypes of bladder cancer cells. In terms of mechanism, it was validated that MCM8 knockdown made Akt, P-Akt, CCND1 and CDK6 levels down-regulated, as well as MAPK9 up-regulated.

**Conclusions:**

Taken together, our study demonstrated an important role of MCM8 in bladder cancer and created a rationale for the therapeutic potential of MCM8 inhibition in human bladder cancer therapy.

## Background

Bladder cancer ranks ninth among prevalent cancers across the world, accounting for a considerable proportion of cancer-related mortalities [1]. The development of bladder cancer includes two primary ways: one is non-muscle-invasive bladder cancer (NMIBC), and the other refers to muscle-invasive bladder cancer (MIBC) [2]. Nowadays, surgical resection is the best treatment option for patients with NMIBC, while multimodal treatment including radical cystectomy combined with neoadjuvant chemotherapy has brought the dawn to MIBC patients [3]. Despite the considerable initial efficacies in these therapeutic approaches, the therapeutic efficacies were seriously limited due to tumor metastasis and resistance of bladder cancer cells to these therapies. Recently, the progress has been made in terms of immunotherapy and targeted therapies for bladder cancer [4, 5], unfortunately, improving the overall survival of patients is still a major clinical challenge. Thus, identifying novel and effective therapeutic targets is essential for gaining a detailed understanding of the pathogenesis of bladder cancer and formulating more efficient molecular therapies strategies. The MCM family, firstly identified in budding yeast *Saccharomyces cerevisiae*, is responsible for DNA replication [6]. MCM2-7 has previously been identified to be involved in a range of carcer types including esophageal squamous cell carcinoma, cervical cancer, gastric cancer and breast cancer, which is a recognized tumor suppressor gene [7–10]. Otherwise, minichromosome maintenance eight homologous recombination repair factor (MCM8) and its physical partner MCM9 are two others newly discovered MCM family members, which have also been shown to be required for DNA replication [11]. Besides, MCM8, homologous to MCM7, participates in DNA extension as a promoter helix [11]. On the other hand, in support of their roles as a tumor promoter, several recent studies have suggested that MCM2-7, MCM8 and MCM10 were overexpressed in hepatocellular carcinoma [12]. However, the specific molecular mechanism of MCM8 in bladder cancer has not been thoroughly understood. Thus, this study was aimed to explore the roles of MCM8 in bladder cancer.

Initial analyses in the present study indicated that the expression level of MCM8 was up-regulated in bladder cancer and positively related to the advanced pathological grade and poor prognosis. Hereafter, in this study, we set out to verify the fact that MCM8 knockdown inhibited cell proliferation and migration, promoted cell apoptosis, arrested cell cycle, as well as suppressed tumor growth. Besides, the mechanism of the effects of MCM8 knockdown on bladder cancer was initially explored. Given our findings and present studies, one could conclude that MCM8 was involved in the development, progression and prognosis of bladder cancer, which implicated the potential of MCM8 as a promising therapeutic target in bladder cancer.

## Methods

### Cell culture

Two bladder cancer cell lines EJ and T24, purchased from Cell Resource Center, Institute of Basic Medicine, Chinese Academy of Medical Sciences (Beijing, China), were selected in the current study, which were cultured in 1640 medium with 10% FBS and incubated at 37 °C, 5% CO_2_ incubator.

### Immunohistochemistry (IHC)

The microarrays (CGt No. XT12-065, Lot No. HBla-Uro105Sur-01) containing 46 bladder cancer tissues and 36 adjacent normal tissues were provided by Shanghai Outdo Biotech Co., Ltd. All patients signed a notification form and provided their relevant clinical characteristics. The study design was approved by Ethics Committee of the Wuxi People’s Hospital Affiliated to Nanjing Medical University. First, the tissue slides were in the oven at 65 °C for 30 min. Then the slides were soaked with xylene and washed with alcohol (China National Pharmaceutical Group Co., Ltd, Beijing, China). Thereafter, the samples were repaired with 1 × EDTA (Beyotime Biotechnology Co., Ltd, Shanghai, China) and blocked with 3% H_2_O_2_ and serum. Next, the MCM8 antibody (1:100, Invitrogen) and second antibody were added to incubate sections at 4 °C overnight. Continuously, the slides were stained with DAB and counterstained with hematoxylin (Baso DiagnosticsInc., Zhuhai, China). Finally, the slides were sealed with neutral resin (China National Pharmaceutical Group Co., Ltd, Beijing, China) and then the images were photographed and analyzed according to the IHC scoring standard. The standard included four categories: negative (0), positive (1–4), ++positive (5–8), or +++ positive (9–12), based on the sum of the staining intensity (varied from weak to strong) and staining extent scores, which graded as 0 (0%), 1 (1–25%), 2 (26–50%), 3 (51–75%), or 4 (76–100%). The IHC score based on the independent identification by three pathologists was used for quantitative analysis. Finally, the high and moderate expression parameters were determined by the median of IHC experimental scores of all tissues.

### Plasmid construction and lentivirus transfection

Three corresponding RNAi target sequences (TGGCAATACATCAGGTGTTAA, CTGGAATTGTCAAAGTCTCAA, AGGCAGCTGGAATCTTTGATT) were designed with MCM8 as templates by Shanghai Bioscienceres Co., Ltd. (Shanghai, China). The specific operation was as follows. The MCM8 target sequences were inserted into the BR-V-108 vector through the restriction sites at both ends and subsequently transformed into TOP 10 E. coli competent cells (Tiangen, Beijing, China). The positive recombinants were screened by the PCR identification and the EndoFree maxi plasmid kit (Tiangen, Beijing, China) was used to extract plasmid according to the manufacturer’s instruction and the plasmid concentration was determined by using a spectrophotometer (Thermo_Nanodrop 2000). The qualified plasmids were transferred to downstream platforms for lentivirus packaging. A three-plasmid BR-V108, BR-V307, BR-V112 co-transfection system was used to collect the 239 T cell supernatant at 48 h and 72 h after transfection and the quality of lentivirus was evaluated. Finally, EJ and T24 cells in logarithmic growth phase were transfected by adding 20 μL 1 × 10^8^ TU/mL lentivirus, following by culturing in 1640 medium with 10% FBS in a 6-well dish with 2 × 10^5^ cells per well. The cell transfection efficiency and knockdown efficiency were evaluated by microscopic fluorescence, qPCR and western blot.

### RNA extraction and qRT-PCR

The total RNA was isolated according to the TRIzol reagent (Sigma, St. Louis, MO, USA) manufacturer’s protocol, and RNA was reverse transcribed to gain cDNA by using the Promega M-MLV Kit (Promega Corporation, Madison, Wisconsin, USA). qRT-PCR was performed using SYBR Green Mastermixs Kit (Vazyme, Nanjing, Jiangsu, China) and the system was 10 μL. The relative expression of mRNA was calculated by the 2^−△△Ct^ method. The primers sequences used in qPCR were as follows (5′-3′): The forward primer of MCM8 was 5′–ATGGCTTTTCTTTGTGCTGC–3′, the reverse primer of MCM8 was 5′–CCAGTCCATCGTAACTGTGAGA–3′. The forward primer of GAPDH was 5′–TGACTTCAACAGCGACACCCA–3′, the reverse primer of GAPDH was 5′–CACCCTGTTGCTGTAGCCAAA–3′.

### Western blot assay

EJ and T24 cells after transfection were collected and lysed with 1 × Lysis Buffer lysis (Cell Signal Technology, Danvers, MA). Also, 10% SDS-PAGE was used to segregate the total proteins and transferred into PVDF membranes followed by blocking with a blocking solution (TBST solution containing 5% skim milk) at room temperature for 1 h. Next, the membranes were incubated with the primary antibodies and second antibodies for 2 h, respectively. After that, the membranes were washed with TBST solution for three times, 10 min each time. Finally, the ECL + plusTM Western blotting system kit was used for color rendering and X-ray imaging was captured. The primary antibodies used in western blotting were as follows: MCM8 (1:1000, Invitrogen), GAPDH (1:3000, Bioworld), Akt (1:1000, CST), P-Akt (1:500, B R&D), CCND1 (1:2000, CST), CDK6 (1:1000, Abcam) and MAPK9 (1:1000, Abcam). The secondary antibody used in western blotting was Goat Anti-Rabbit (1:3000, Beyotime).

### MTT assay

EJ and T24 cells, after transfected with lentivirus, were digested and resuspended into the cell suspension at the density of 2000 cells/well. 100 μL cell suspension was cultured in 96-well plates and determined for 5 days. Each well contained three repetitions. 20 μL MTT (5 mg/mL) and 100 μL DMSO were added into 96-well plates. OD value at 490 nm wave length was detected with microplate reader (Tecan infinite, Mannedorf Zurich, Switzerland).

### Cell migration assay

To evaluate the ability of cell migration, we used the wound-healing assay and the transwell assay. EJ and T24 cells, which were transfected and collected, were seeded into a 96-well plate at the density of 5 × 10^4^ cells/well. Then, the cells were incubated in an incubator with 5% CO_2_ at 37 °C. The cells were observed and photographed by a microscope at 0 h, 4 h and 8 h. The experiment was repeated three times and the migration rate of cells was evaluated based on the scratch images.

In terms of the transwell assay, the EJ and T24 cells, which were transfected with lentivirus, were prepared at the density of 5 × 10^4^ cells/mL and loaded into the upper chamber containing serum-free medium. Then, the upper chamber was transferred to the lower chamber with medium containing 30% FBS and incubated for 72 h. After that, 400 µL Giemsa was added for cells staining and the cell migration ability was quantified.

### Cell apoptosis assay

Lentivirus-transfected EJ and T24 cells were cultured in 6-well plates (2 mL/well) for 5 days. 5 μL Annexin V-APC was added for staining 10–15 min at room temperature in the dark. After that, the cell suspension was centrifuged at 1500 rpm for 3 min, and then 100 μL of 1 × binding buffer was added to resuspend the cells. Finally, 5μL of PI staining solution was added. The cell apoptosis level was measured by using FACSCalibur (BD Biosciences, San Jose, CA, USA) to assess the apoptotic rate.

### Human apoptosis antibody array

The Human Apoptosis Antibody Array was performed to explore the effects of MCM8 knockdown on the apoptosis-related protein expression in T24 cells. After the cells were lysed, the Handling Array membranes were blocked by 2 mL 1 × Wash Buffer II and incubated with cell lysates and 1 × Biotin-conjugated Anti-Cytokines overnight at 4 °C. Finally, the signals of membranes were tracked by chemiluminescence imaging system.

### The construction of nude mouse tumor formation model

The animal study was approved by Ethics Committee of the Wuxi People’s Hospital Affiliated to Nanjing Medical University.T24 cells transfected with shCtrl or shMCM8 lentiviruses were subcutaneously injected into the four-week-old female BALB-c nude mice from Shanghai Lingchang Biological Technology Co., Ltd. (Shanghai, China) to construct the Xenograft models, each group containing five mice. During the feeding period, the tumor volume was measured. On the last day of feeding, 0.7% sodium pentobarbital was injected intraperitoneally for several min, and the fluorescence was observed by the in vivo imaging system (IVIS Spectrum, Perkin Elmer). After 40 days, the mice were sacrificed using cervical dislocation to collect tumors. The tumors were weighed and photographed, and finally frozen in liquid nitrogen and stored at − 80 °C.

### Ki-67 staining

The tumor tissues from mice were fixed with 4% paraformaldehyde and 0.3% TritonX-100. Then, the slides were incubated with primary antibody Ki-67 (1:200, Abcam, CA, USA) at 4 °C overnight in the dark and the secondary antibody goat anti-rabbit IgG H&L (HRP) (1:400, Abcam, CA, USA) was added as above. Finally, the slides were stained by Hematoxylin and Eosin (Baso, Zhuhai, Guangdong, China), and observed by a microscope.

### Statistical analysis

Data in this study were analyzed by GraphPad Prism 8 (San Diego, CA, USA) and SPSS 19.0 (IBM, SPSS, Chicago, IL, USA), and presented as the mean ± SD. Student’s *t*-test and one-way ANOVA were used to analyze the statistical significance. The Pearson correlation analysis and Mann–Whitney *U* analysis were used to assess the relationship between the expression of MCM8 and characteristics of bladder cancer. *P* < 0.05 was considered to be significantly different. All the experiments were in triplicate.

## Results

### The expression pattern and significance of MCM8 in bladder cancer

**Fig. 1:**
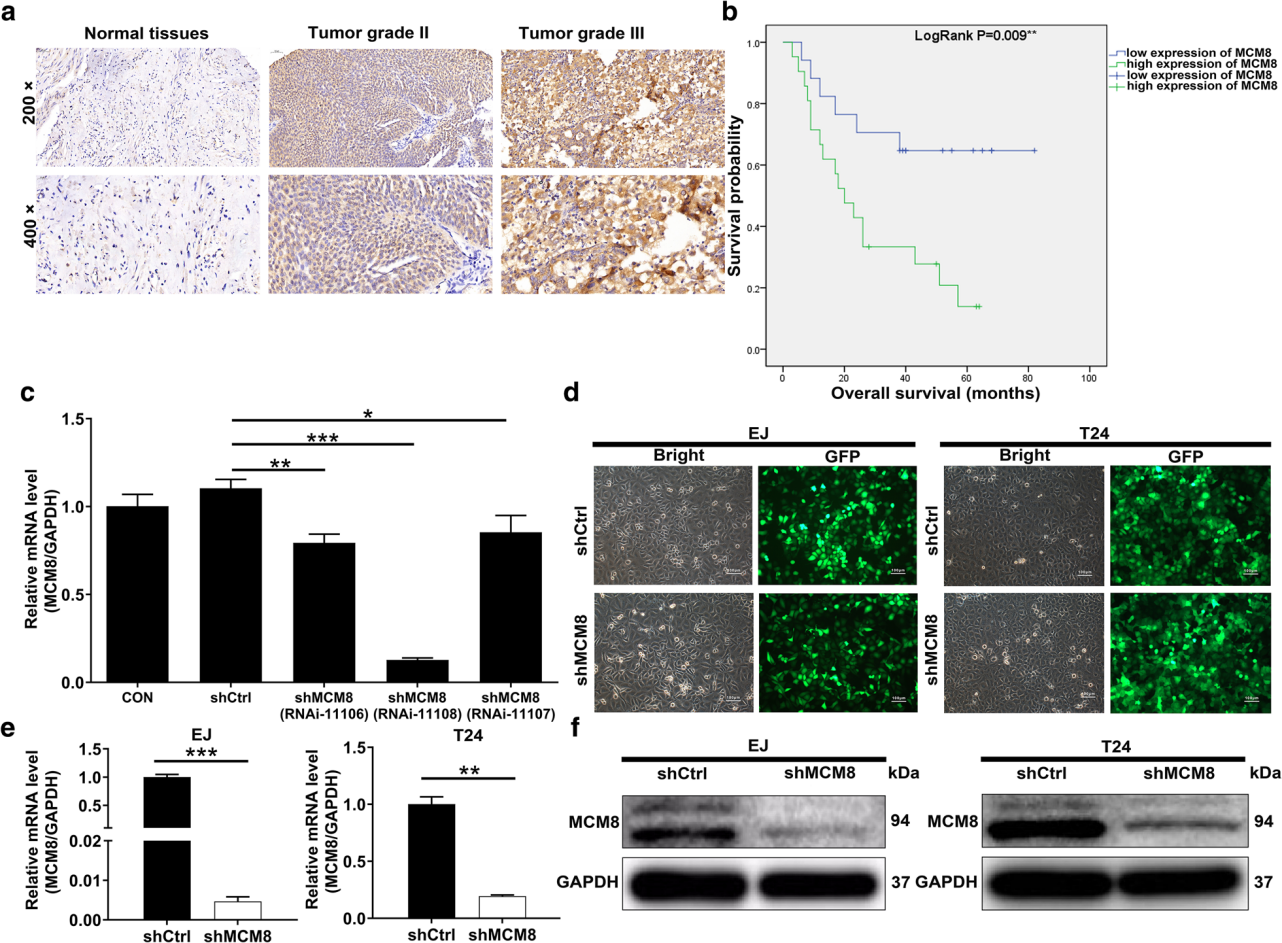
MCM8 was up-regulated in bladder cancer and MCM8 knockdown cell model was constructed. **a** The expression levels of MCM8 in bladder cancer tumor tissues and para-carcinoma tissues were determined by immunohistochemical staining. **b** Kaplan–Meier survival analysis was performed to reveal the relationship between MCM8 expression and prognosis of bladder cancer patients. **c** The knockdown efficiencies of MCM8 in shMCM8 group were detected by qRT-PCR. **d** The fluorescence expression in cells was observed after 72 h-transfection. Magnification times: 200×. **e** The MCM8 mRNA expression in bladder cancer cell lines after transfection was analyzed by qRT-PCR. **f** The expression of MCM8 protein in bladder cancer cell lines after transfection was detected by western blot. Results were presented as mean ± SD. *P < 0.05, **P < 0.01, ***P < 0.001

Initially, the differential expression of MCM8 analyses were performed on bladder cancer tumor tissues and adjacent normal tissues, which revealed 52.2% of bladder cancer tumor tissues with high MCM8 levels, while low MCM8 expression in all normal tissues (*P* < 0.001, Table 1, Fig. 1a). Subsequently, the correlation between MCM8 expression and clinical characteristics was evaluated using the Mann–Whitney *U* analysis and the Pearson correlation analysis, which demonstrated that MCM8 expression was positively correlated with pathological grade with a *P* value of 0.001 (Table 2, Table 3), yet no significant correlation was observed between MCM8 expression and age, gender, tumor size, lymphadenopathy, stage or T Infiltrate (Table 2). Survival curves according to Kaplan–Meier showed that MCM8 expression was significantly linked to the overall survival (Fig. 1b). To summarize, we reasoned that MCM8 was extensively associated with the development, progression and prognosis of bladder cancer.

**Table 1:** Expression patterns of MCM8 in bladder cancer tissues and para-carcinoma tissues revealed in immunohistochemistry analysis

MCM8 expression	Tumor tissue		Para-carcinoma tissue		*P* value
	Cases	Percentage	Cases	Percentage	
Low	22	47.80	36	100	< 0.001
High	24	52.20	0	–

**Table 2:** Relationship between MCM8 expression and tumor characteristics in patients with bladder cancer

Features	No. of patients	MCM8 expression		*P* value
		low	high	
All patients	46	22	24	
Age (years)				0.559
< 74	23	10	13	
≥ 74	23	12	11	
Gender				0.056
Male	39	21	18	
Female	7	1	6	
Tumor size				1.000
< 4 cm	18	9	9	
≥ 4 cm	26	13	13	
Lymphadenopathy				0.206
Yes	4	1	3	
No	27	16	11	
Grade				0.001
II	16	13	3	
III	30	9	21	
Stage				0.334
1	4	2	2	
2	8	5	3	
3	12	7	5	
4	5	1	4	
T Infiltrate				0.162
T1	8	5	3	
T2	12	7	5	
T3	16	6	10	
T4	3	1	2

**Table 3:** Relationship between MCM8 expression and tumor characteristics in patients with bladder cancer

		MCM8
Grade	Pearson correlation	0.489
	Signification (double-tailed)	0.001
	N	46

### Construction of MCM8 knockdown cell models

In order to evaluate the biological implications of MCM8 in bladder cancer progression, T24 cells were transfected with RNAi targeting MCM8 (RNAi-11106, RNAi-11107, RNAi-11108), three of which successfully reduced the expression of MCM8 as evidenced by qRT-PCR analysis, especially RNAi-11108 (Fig. 1c). As a result, RNAi-11108 was chosen for further experimentation. It followed that a > 80% efficiency of transfection was obtained through observing the green fluorescent protein (GFP) in EJ and T24 cells (Fig. 1d). Next, we determined the knockdown efficiencies of MCM8 in EJ and T24 cells via qRT-PCR and western blot analysis. As the results are shown in Fig. 1e, f, MCM8 mRNA and protein expression was significantly decreased. The behavior of the all above data made us conclude that MCM8 knockdown cell models were successfully constructed.

### Loss-of-function of MCM8 attenuated cell proliferation and migration of bladder cancer cells in vitro

**Fig. 2:**
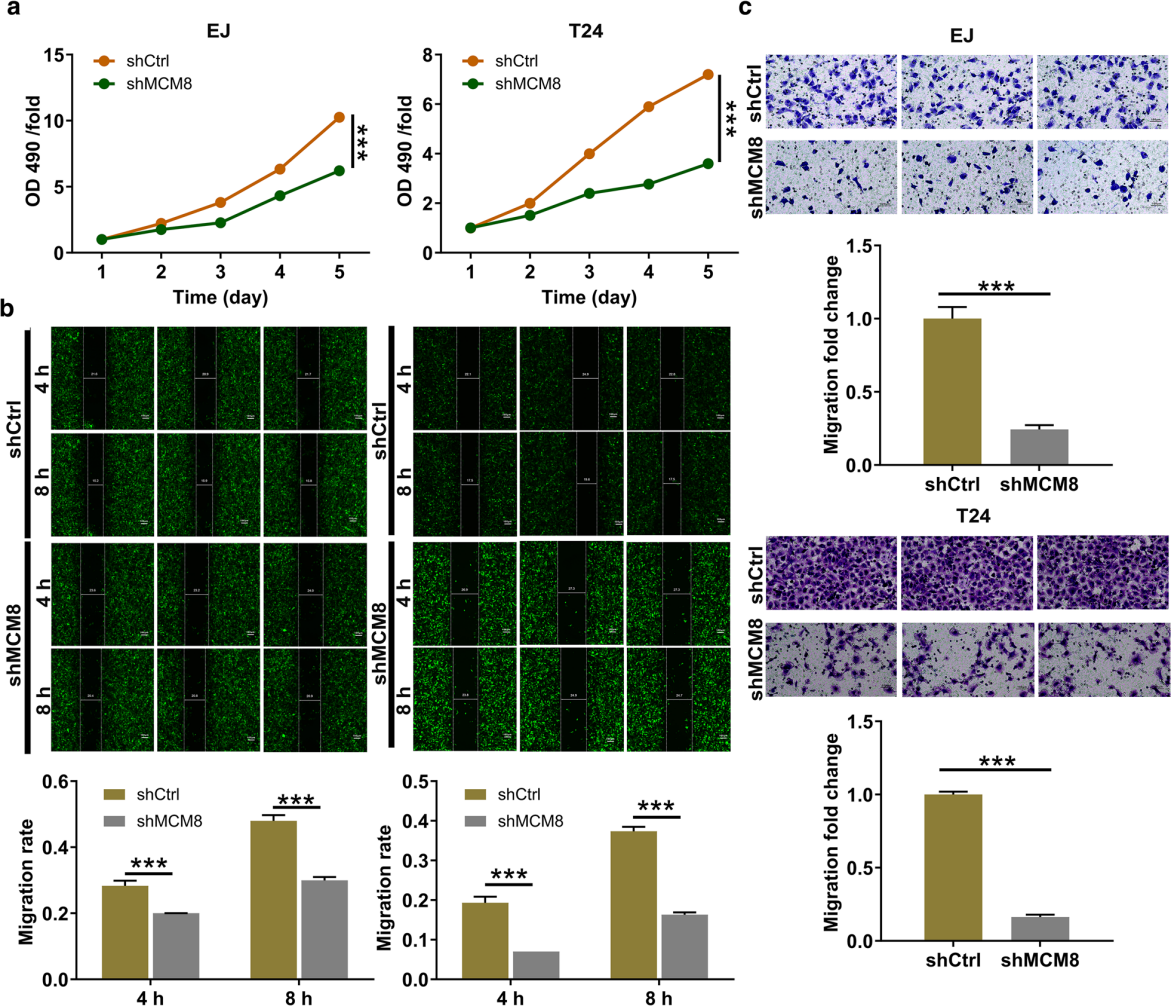
Loss-of-function of MCM8 attenuated cell proliferation and migration of bladder cancer cells. **a** The cell proliferation rate was evaluated in bladder cancer cell lines after transfection by the MTT assay. **b** The migration rate of cells was detected in bladder cancer cell lines after transfection by wound-healing assay. **c** The migration rate of cells was detected in bladder cancer cell lines after transfection by transwell assay. Results were presented as mean ± SD. ***P < 0.001

Followed by the transfection with RNAi-11108, the MTT assay, the wound-healing assay, coupled with the transwell assay were exploited to evaluate the cell proliferation and migration in vitro. The MTT assay showed significantly lower cell proliferation level in shMCM8 cells relative to shCtrl cells (*P* < 0.001, Fig. 2a). Simultaneously, as depicted in the resulting wound-healing assay, the larger intervals between the cells in shMCM8 groups at the leading edge were observed, indicating that the cell migration ability was attenuated (*P* < 0.001, Fig. 2b). Subsequently, a transwell assay was performed to verify that the cell migration ability was inhibited by MCM8 knockdown (*P* < 0.001, Fig. 2c). Overall, these results suggested that the cell proliferation and migration abilities were all attenuated due to the loss-of-function of MCM8.

### Loss-of-function of MCM8 ameliorated cell cycle and apoptosis of bladder cancer cells in vitro

**Fig. 3:**
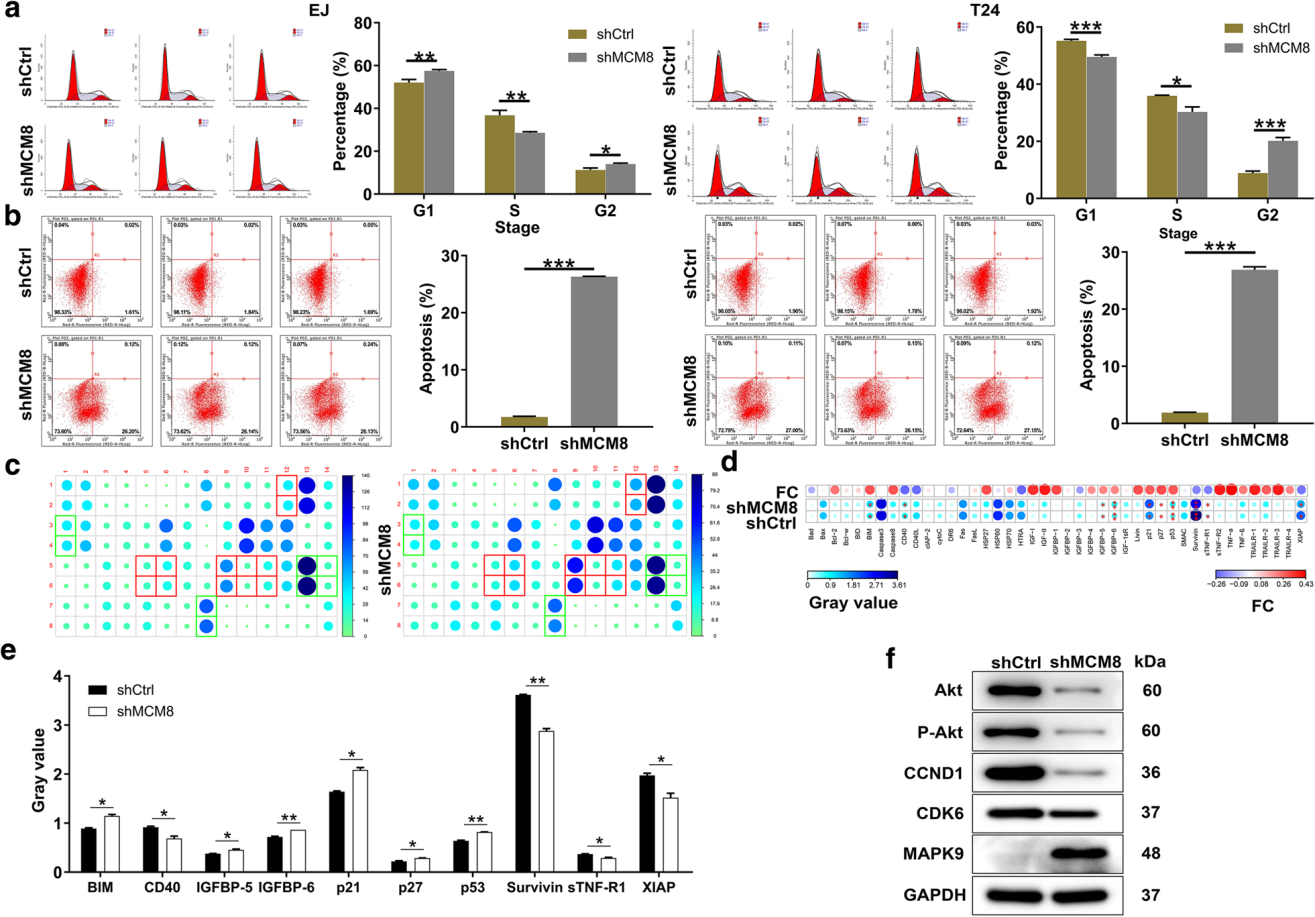
Loss-of-function of MCM8 ameliorated cell cycle and apoptosis of bladder cancer cells. **a** The effects of MCM8 knockdown on cell cycle were determined by flow cytometry. **b** The effects of MCM8 knockdown on cell apoptosis were examined by flow cytometry. **c** The expression of apoptosis-related proteins in T24 cells transfected with shMCM8 was measured by ECL with Human Apoptosis Antibody Array. The results circled in red indicated that the protein expression was up-regulated and *P* < 0.05. **d** Protein expression was presented in grayscale and visualized by R studio. **e** The expression levels of apoptosis-related proteins were analyzed in T24 cells with shMCM8. **f** The expression of Akt, P-Akt, CCND1, CDK6 and MAPK9 was detected by western blot. Results were presented as mean ± SD. *P < 0.05, **P < 0.01, ***P < 0.001

In an attempt to identify the effects of MCM8 knockdown on cell cycle progression and cell apoptosis, the flow cytometry analysis was performed. It could be seen in part a of Fig. 3 that the shMCM8 group of both cells exhibited a decreased cell population in S phase, reversely, an increased arrest in G2 phase. Additionally, the apoptosis percentage increased in shMCM8 group in comparison with shCtrl group (*P* < 0.001, Fig. 3b). Next, the western blot analysis was performed to evaluate expression patterns of apoptosis-related factors. As expected, the expression of BIM (BCL2L11, BCL2-like 11), IGFBP-5 (insulin like growth factor binding protein 5), IGFBP-6 (insulin like growth factor binding protein 6), p21 (21 kDa protein), p27 (27 kDa protein) and p53 (53 kDa protein) was augmented, while those of CD40 (CD40 molecule, TNF receptor superfamily member 5), Survivin (BIRC5, baculoviral IAP repeat containing 5), sTNF-R1 (tumor necrosis factor receptor superfamily, member 1a) and XIAP (X-linked inhibitor of apoptosis) was decreased after silencing MCM8 (Fig. 3c–e). Taken together, these results showed that silencing of MCM8 arrested the cell cycle of bladder cancer cells while accelerating their apoptosis. Furthermore, some cancer-related factors were detected by the western blot assay, accompanied by the down-regulation of Akt (protein kinase B, PKB), P-Akt (phosphorylated AKT), CCND1 (cyclin D1) and CDK6 (cyclin-dependent kinase 6) levels, as well as the up-regulation of MAPK9 (mitogen-activated protein kinase 9) in shMCM8 group (Fig. 3F).

### Loss-of-function of MCM8 curbed bladder cancer tumorigenesis in vivo

**Fig. 4:**
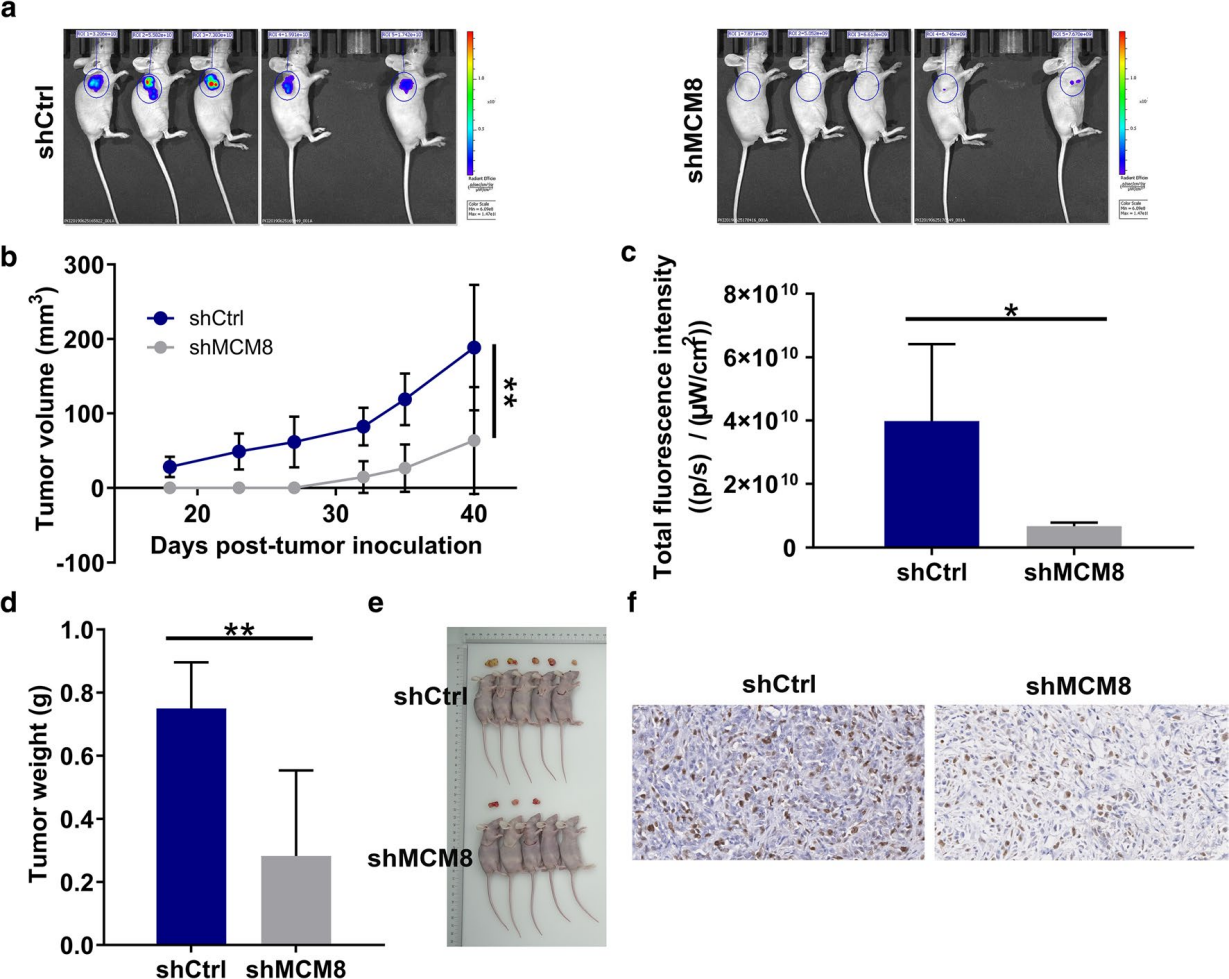
Loss-of-function of MCM8 curbed bladder cancer tumorigenesis. **a** A nude mice model of MCM8 knockdown was constructed. **b** The volume of tumors was tested from feeding to sacrifice. **c** The fluorescence intensity was obtained through injection of D-Luciferase before sacrificing the mice. **d** The weight of tumors was measured after sacrificing mice. **e** The photograph of tumors was taken after removing tumors. **f** The value of Ki-67 was detected by IHC in tumor sections. Magnification times: 400 × . Results were presented as mean ± SD. *P < 0.05, **P < 0.01

Lastly, nude mice were subcutaneously injected with T24 cells transfected with shCtrl or shMCM8 lentiviruses to establish a subcutaneous xenograft model, so as to further demonstrate the tumor-suppressive effects of MCM8 in vivo (Fig. 4a). As shown in Fig. 4b, the volume of transplanted tumors was reduced by MCM8 knockdown (*P* < 0.01). Under in vivo imaging, the decreased fluorescence was considered to be impaired tumor growth in shMCM8 group (*P* < 0.05, Fig. 4c). After sacrificing mice, the tumors of nude mice were then harvested and assessed, and the weight was found to be lighter in shMCM8 group (*P* < 0.01, Fig. 4d, e). At the same time, the expression patterns of factors related to proliferation were determined in tumor tissues. The results showed that the absence of MCM8 led to diminished expression of Ki-67 (Fig. 4f). All in all, these data suggested that lentivirus shMCM8 was successfully transferred to T24 cells, inhibiting their growth as xenografts in nude mice.

## Discussion

Bladder cancer is one of the fatal cancers of the urinary system and one of the highest recurrence rates of all cancers, as well as the ninth most common cancer worldwide [13]. Even though around 75% of the newly diagnosed cases are non-muscle-invasive bladder carcinomas (NIMBC) with good prognosis and survival rate, patients with muscle-invasive bladder cancer (MIBC) have a very poor prognosis [14]. On the other hand, there has been limited progress in exploring appropriate therapies to elevate survival rate of patients with MIBC to date. Clearly, it is imperative to explore new therapeutic interventions, to improve the overall response rate and subsequent survival rate of MIBC patients.

MCM8, as one of the DNA replication licensing factors, conducts a vital role in the initiation and extension of DNA replication [15]. An enormous number of studies implicated that MCM8 was recruited into DNA repair sites to promote DNA homologous recombination and double-strand breaks [16]. In view of the fact that cancer cells are under more pressure to replicate than normal cells due to the high growth stimulation of carcinogenesis [17, 18], some studies point out that MCM8 played an essential role in the repair process of replication stress [19]. Beyond its role in DNA replication, it was reportedly indicated that MCM8 was related to the invasion and metastasis characteristics of several human tumors. Existing study elucidated that MCM8 amplification and overexpression underlined the aggressiveness of prostate cancer [20]. More importantly, the up-regulation of MCM8 was regarded as a valuable independent prognostic indicator in patients with pancreatic cancer, suggesting a shorter overall survival time [21]. However, the influence of MCM8 on bladder cancer remains far from being fully elucidated.

Here we demonstrated that MCM8 was up-regulated in bladder cancer. The enhanced expression of MCM8 was found to be associated with the advanced histologic stage of malignant tumors and poor prognosis of patients. Furthermore, inhibition of MCM8 in human bladder cancer cell lines EJ and T24 decreased cell proliferation and migration, as well as induced apoptosis, indicating that MCM8 might be a potential therapeutic target in bladder cancer. Next, expression patterns of apoptosis-related factors were detected. As expected, the expression of BIM, IGFBP-5, IGFBP-6, p21, p27 and p53 was augmented, while those of CD40, Survivin, sTNF-R1 and XIAP were decreased after silencing MCM8. Here we also demonstrated that the inhibition of MCM8 decreased the number of advanced tumors in mice.

We further elucidated that MCM8 knockdown made Akt, P-Akt, CCND1 and CDK6 levels down-regulated, as well as MAPK9 up-regulated. Published research manifested that DANCR (differentiation antagonizing non-protein coding RNA) promoted metastasis and proliferation in bladder cancer cells by enhancing IL-11-STAT3 (interleukin-11-signal transducers and activators of transcription-3) signaling and CCND1 expression [22]. Additionally, it is known that CDK6 is a member of the cell cycle dependent kinase (CDK) family and is an important regulator of cell cycle progress, especially plays an important role in the progress of G1 phase and the transition of G1/S phase [23]. Li et al*.* reported that DDX11-AS1 (DEAD/H box protein 11 antisense RNA 1) exacerbated bladder cancer progression by enhancing CDK6 expression via suppressing miR-499b-5p [24]. Besides, ERK/MAPK (extracellular signal-regulated kinase/mitogen-activated protein kinase) pathway was reported to be a major signaling pathway involved in cell growth and proliferation [25]. Akt is known for its mechanistic roles in cell growth, proliferation, survival and metabolism. The results from Li et al*.* demonstrated that ZSCAN16 (Zinc Finger and SCAN Domain Containing 16) promoted proliferation, migration and invasion of bladder cancer cells via regulating NF-kB (nuclear factor kappa-light-chain-enhancer of activated B cells), AKT and mTOR (mechanistic target of rapamycin kinase) [26]. Additionally, RASAL2 (RAS protein activator like 2) inhibited tumor angiogenesis via p-AKT/ETS1 (ETS proto-oncogene 1, transcription factor) signaling in bladder cancer [27]. As such, it could be speculated that the anti-tumorigenic effects of MCM8 inhibition on bladder cancer was primarily mediated by those proteins.

## Conclusion

In conclusion, our collective findings demonstrated that MCM8 inhibition contributed to the suppression of bladder cancer tumorigenesis, laying the groundwork for potential therapeutic target to curb carcinogenesis.

## Data Availability

All data generated or analysed during this study are included in this published article.
